# Effect of local TGF-β1 and IGF-1 release on implant fixation: comparison with hydroxyapatite coating: A paired study in dogs

**DOI:** 10.3109/17453670903153519

**Published:** 2009-08-01

**Authors:** Anders Lamberg, Joan E Bechtold, Jørgen Baas, Kjeld Søballe, Brian Elmengaard

**Affiliations:** ^1^Orthopaedic Research Laboratory, Department of Orthopaedic Surgery, Aarhus University HospitalDenmark; ^2^Orthopaedic Biomechanics Laboratory, Midwest Orthopaedic and Minneapolis Medical Research FoundationsMinneapolis, MNUSA

## Abstract

**Background and purpose** Hydroxyapatite (HA) coating stimulates the osseointegration of cementless orthopedic implants. Recently, locally released osteogenic growth factors have also been shown experimentally to stimulate osseointegration so that bone fills gaps around orthopedic implants. Here, we have compared the effect of local release of TGF-β 1 and IGF-1 with that of hydroxyapatite coating on implant fixation.

**Method** Weight-bearing implants with a 0.75-mm surrounding gap were inserted bilaterally in the knees of 10 dogs. Growth factors were incorporated in a biodegradable poly(D,L-lactide) coating on porous coated titanium implants. Plasma-sprayed HA implants served as controls. The dogs were killed at 4 weeks and the implants were evaluated by mechanical push-out test and by histomorphometry.

**Results** There was no difference in any of the mechanical parameters. Bone ongrowth was 3-fold higher for HA-coated implants (p < 0.001). For growth factor-coated implants, bone volume was 26% higher in the inner half of the gap and 28% higher in the outer half compared to HA (p < 0.03).

**Interpretation-** The mechanical fixation of porous-coated titanium implants with local growth factor release is comparable to that of HA coating. While HA mainly stimulated bone ongrowth, local release of TGFβ 1 and IGF-1 stimulated gap healing.

## Introduction

The long-term fixation of cementless total hip replacements (THRs) is positively affected by rapid bony integration. This has been confirmed by RSA studies that have concluded that early implant migration is a strong predictor of early loosening (Karrholm et al. 1994, Hauptfleisch et al. 2006).

Implants with a hydroxyapatite (HA) coating stimulate bone ingrowth to implants. The highly osteoconductive nature of HA coatings has been demonstrated experimentally (Soballe et al. 1990, 1992), and superior longevity has been demonstrated clinically (Soballe et al. 1993, Capello et al. 2006, Kelly et al. 2006). Recently, new adjuvant therapies using osteogenic growth factors have been introduced. Experimental studies have shown that local growth factor treatment can stimulate osseointegration and gap healing of orthopedic implants (Lamberg et al. 2006, Sumner et al. 2006). Some of these treatments have also proven useful in fracture healing and spinal fusion (Schmidmaier et al. 2001a, Kandziora et al. 2002).

We have previously shown that the combination of transforming growth factor β1 (TGF-β1) and insulin-like growth factor 1 (IGF-1) in a biodegradable poly(D,L-lactide) coating markedly increases the fixation and osseointegration of porous coated titanium implants in an unloaded 1-mm gap model (Lamberg et al. 2006). To follow up on the comparison with the negative control group (Ti), we next compared local growth factor treatment pairwise with HA coating, a known effective stimulator of osseointegration.

In a paired study using dogs, we compared porous-coated implants with 2 different coatings applied to the base titanium alloy, in a loaded 0.75-mm gap model in the knee. In one group, implants were coated with a biodegradable carrier, poly(D,L-lactide), containing a combination of TGF-β1 and IGF-1. In the other group, the implants were plasma-sprayed with hydroxyapatite coating. We hypothesized that the growth factor coating would result in better mechanical fixation and more bone on and around the implant.

## Material and methods

### Study design

**Figure 1.**
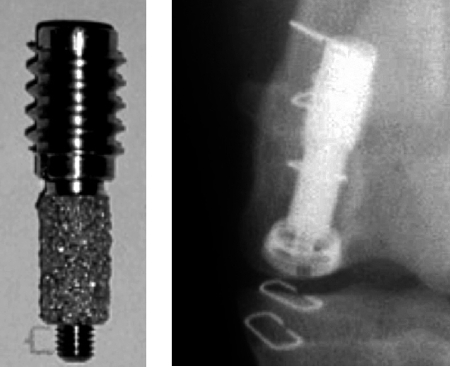
Illustrations of the model. The polyethylene plug transferring load from the tibial cartilage to the implant is not shown. The implant is surrounded by a 0.75-mm gap, and it is loaded during each gait cycle. In addition, there is a constant flow of joint fluid passing the surface. The HA-coated implants were inserted in the left femur and the growth factor-coated implants in the right femur.

Following approval of the institutional Animal Care and Use committee, we performed a paired study in 10 skeletally mature purpose-bred hound dogs with a mean weight of 22 (SD 1.2) kg. We used an established model (Soballe et al. 1992) in which the implants are weight bearing and surrounded by a circumferential gap of 0.75 mm (Figure 1). Implants were inserted in the distal femur, in a cancellous bone site oriented along the medial condyles' major weight-bearing axis. The growth factor-coated implants were inserted in the right femoral condyle, and the HA-coated control implants were inserted in the left femoral condyle. The observation period was 4 weeks.

### Coatings

The coatings were applied to cylindrical, porous-coated titanium (Ti-6Al-4V) implants measuring nominally 6 mm × 10 mm (Biomet Inc., Warsaw IN). In our laboratory, we mixed sterile filtered poly(D,L-lactide) (Resomer 203; Boehringer Ingelheim, Germany) and carrier-free recombinant human TGF-β1 and IGF-1 (R&D Systems, Minneapolis MN) in ethyl acetate to a w/w ratio of 1% for the TGF-β1 and 5% for the IGF-1. The implants were dipped in the solution and air-dried for 1 min before being dipped again. Based on earlier work, we estimated the coating to be 10–20 microns thick and that each implant contained 28 µg TGF-β1 and 140 µg IGF-1 (Lamberg et al. 2006). The control implants received a plasma-sprayed HA coating with a thickness of approximately 40–70 µm (Biomet).

### Surgery

We performed the surgery with the dogs under general anesthesia. The incision was made medial to the patella, and the most distal point of the medial femoral condyle was identified. 7.5-mm holes were drilled in the medial femoral condyle. A stabilizing anchor screw with a central threaded pin was first inserted, and then the implant was screwed onto the piston of the anchor screw. This resulted in a 0.75-mm circumferential gap around the implant. A centralizing ring and polyethylene plug was mounted to secure the position of the implant concentric within the gap (Figure 1). The polyethylene plug extended 1 mm into the joint, transmitting load to the implant in each gait cycle or knee flexion. The soft tissue was closed in layers. All animals were fully weight-bearing within 24 h. The animals received the antibiotic cefuroxime (Rocephin, Roche Pharmaceutical) prophylactically (1 g intravenously before surgery and 1 g intramuscularly daily for 3 days). The dogs were evaluated daily by the institutional veterinarian.

### Preparation

Pending preparation, the bone-implant specimens were kept frozen at –20°C. To separate the specimens for mechanical testing from those for histomorphometry, the bone-implant specimens were cut transversely with a water-cooled diamond band saw. The outermost 3.5 mm was used for mechanical testing and the remaining 6.5 mm was used for histomorphometry. The latter was dehydrated in graded ethanol (70–100%) with 0.4% basic fuchsin and then embedded in methylmethacrylate.

### Mechanical testing

Implants were tested to failure by a push-out test on an Instron Universal Test Machine (Model 4302; Instron, High Wycombe, UK). The specimens were placed on a metal support jig with a 7.4-mm circular opening. A preload of 2 N was applied, to ensure contact with the implant. The displacement rate was 5.0 mm/min. Maximum shear strength (MPa), apparent shear stiffness (MPa/mm), and energy to failure (J/m2) were calculated from the recorded load-displacement data. The analysis was performed in blinded fashion.

### Histomorphometry

**Figure 2.**
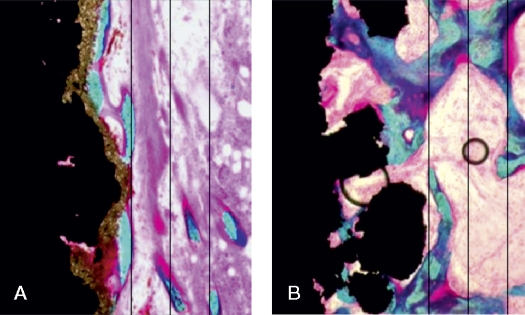
Images from histomorphometry. A. hydroxyapatite-coated implant. B. Growth factor-coated implant. The lines indicate the inner and outer zones where the tissue volume is measured. Ongrowth is defined as tissue in direct contact with the implant surface or the hydroxyapatite coating.

The specimens underwent random vertical sectioning on a Leiden microtome (Leiden, Holland), and were counterstained with 2% light green (Overgaard et al. 2000). The analyses were done with Cast grid software (Olympus). The obvious presence of hydroxyapatite on the control implants made true blinding impossible, but the examiner did not know which implant was the pair match. We quantified bone and fibrous tissue by stereological principles (Gundersen et al. 1988). The ongrowth was estimated using line interception technique and the gap volume fractions were estimated by point interception technique. The gap was divided into 2 zones: the inner zone adjacent to the implant surface, and the outer zone adjacent to the edge of the drill hole (Figure 2).

### Statistics

As an aid in designing this study, we performed a sample-size calculation based on the assumption that the mean change is 50% and an SD of the change is 50%. The primary endpoint for the sample-size calculation was the maximum shear strength. With a risk of type 1 error of 5% and a power of 80%, we needed 8 animals in the study. In order to allow for exclusions due to animal deaths, mistakes in preparation of specimens, and other unforeseen incidents, this number was increased. Stata 8.0 software (StataCorp, College Station, TX) was used for the statistical calculations. The paired data were analyzed by the paired t-test. We considered two tailed p-values less than 0.05 to be statistically significant. All data are presented as mean with 95% confidence interval (CI).

## Results

All dogs were fully mobilized within 24 h and no signs of infections were observed. Cultures taken from the implantation site at retrieval were all without bacterial growth. We had to exclude 2 implant pairs from the mechanical testing, 1 due to a surgical malalignment of the implant and 1 due to a technical error during preparation.

**Figure 3.**
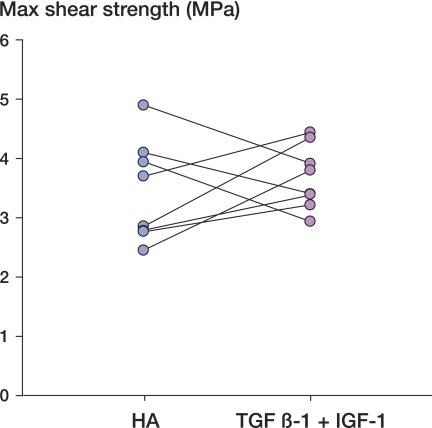
Line plot of maximum shear strength. Paired data are shown interconnected. (p = 0.5).

The mechanical push-out test found all implants to be well fixed, with shear strength ranging from 2.5 to 5 MPa. There were no statistically significant differences in any of the 3 mechanical parameters (Figure 3 and Table 1).

**Table 1.** Mechanical data (n = 8; 2 excluded). No difference was seen in any of the mechanical parameters (p ≥ 0.23). The data are presented as mean (95% CI)

	Energy to failure J/m^2^	Shear stiffness Mpa/mm
Hydroxyapatite	561 (340–781)	19.9 (16.2–23.5)
TGF-β1 and IGF-1	713 (585–842)	18.7 (16.0–21.5)

**Figure 4.**
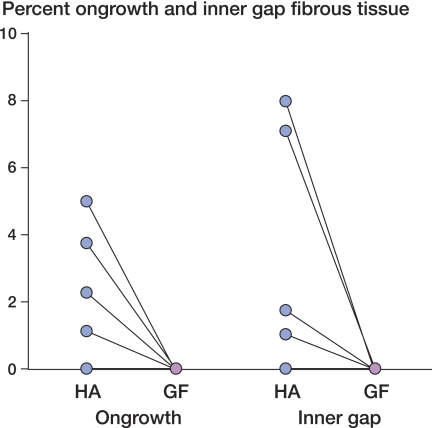
Graph showing percentage of fibrous tissue on the implant surface (ongrowth), and in the inner gap (p = 0.05). Paired data are shown interconnected. HA: Hydroxyapatite, GF: growth factor.

**Figure 5.**
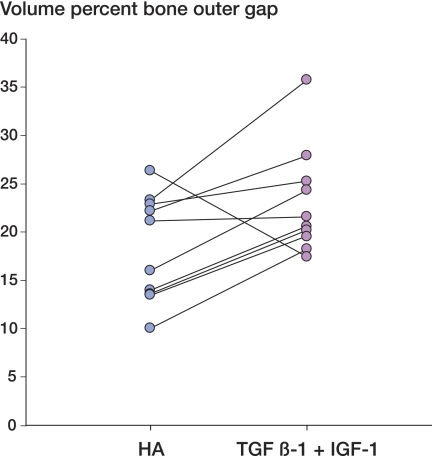
Graph showing the data for the outer zone bone volume (p = 0.03). Paired data are shown interconnected.

In contrast to the push-out test, the histomorphometric analysis showed differences in tissue distribution around the implants. Bone ongrowth to the implants was 3-fold higher for HA-coated implants (p < 0.001) (Table 2). There was ongrowth of fibrous tissue in 4 of the 10 HA-coated implants (up to 8%), and no fibrous tissue on the growth factor-coated implants (p = 0.05) (Figure 4). In the inner half of the gap, the bone volume fraction was 26% higher in the growth factor-coated implant group (p = 0.01) (Table 2). In the outer half of the gap, the bone volume fraction was 28% higher in the growth factor-coated implant group (p = 0.03) (Figure 5). There were no signs of residual polymer on the implant surfaces.

**Table 2.** Histomorphometric data for bone. Data are presented as mean (95% CI) percentage

	Ongrowth	Inner gap
TGF−β1 and IGF-1	14 (11–16)	29 (27–32) **^b^**
Hydroxyapatite	44 (40–48 ) **^a^**	23 (19–27)

**^a^** p < 0.001; **^b^** p = 0.01.

## Discussion

In this study we compared the effects of local release of TGF-β1 and IGF-1 with that of a hydroxyapatite coating on the fixation of weight-bearing implants surrounded by a circumferential gap. Although we did not find any difference in the mechanical fixation, the effect on the surrounding tissue was different with the two interventions. While the growth factors primarily stimulated bone in the gap, the HA coating had superior bone ongrowth.

We used an experimental animal model that attempts to mimic the cancellous bone fixation of clinical implants. The implants are weight-bearing and their intraarticular location results in a flow of synovial fluid along the implant interface. The main idea of this model is to test whether a treatment will enhance the initial implant fixation, and thus prevent early loosening. The observation period of 4 weeks has been shown to detect differences in early fixation in this model (Soballe et al. 2003, 2004). We used a paired design, thus eliminating the inter-individual variation between animals.

As with all other experimental models, this model also has limitations. The loading is mainly in shear and does not include the rotational forces that also apply to human implants. The dog has a bone turnover which is 2–3 times higher than that in humans. This may influence the effect of certain treatments. Moreover, the implants are simplified in shape and size and do not include fixation points of both cancellous and cortical bone. However, this allows implantation in a highly controlled environment and permits unbiased histological evaluation.

The histological analysis showed that the solid fixation in both groups was obtained in a different way for each group: the HA-coated implants had a greater surface bone ongrowth, whereas the growth factor-coated Ti implants had greater new bone formation in the surrounding gap.

The effect of local release of TGF-β1 and IGF-1 on the tissue response was different than for HA. In accordance with previous studies (Lamberg et al. 2006), no fibrous tissue ongrowth was found on any implant with TGF-β1 and IGF-1. Without adjuvant therapy, a high degree of fibrous tissue anchorage would be expected on titanium implants in this implant model (Soballe et al. 1992, Elmengaard et al. 2005). We believe that the elimination of fibrous tissue on the interface is not an effect related to the poly(D,L-lactide) scaffold. A parallel study has shown no difference in fibrous tissue ongrowth between poly(D,L-lactide)-coated and control titanium implants (unpublished data). To our knowledge, no other adjuvant treatment has been shown experimentally to stimulate gap healing around titanium implants better than HA.

Applications combining 2 or more growth factors may be more favorable due to additive or synergistic effects on bone formation. TGF-β1 and IGF-1 are both highly expressed during bone growth (Dequeker et al. 1993, Nicolas et al. 1994, Lammens et al. 1998, Eingartner et al. 1999, Pfeilschifter et al. 1999, Kveiborg et al. 2001). A synergistic effect of TGF-β1 and IGF-1 on bone healing has been reported (Schmidmaier et al. 2003). Our study does not allow us to make conclusions on the contribution of each component to the result. However, the overall effect of the combined growth factors was higher compared to historic data from studies using the same model and single administration of TGF-β1 (Lind et al. 1996a, b).

We used a polymer for controlled release of the growth factors. The poly(D,L-lactide) has been used successfully as a drug carrier (Schmidmaier et al. 2001b, Rose et al. 2004). Polylactides are also used to make screws and plates for fracture treatment, and are generally considered safe. This delivery system has advantages over other methods such as simple surface adsorption of growth factors and collagen sponges, as it has been documented to be mechanically stable (Schmidmaier et al. 2001b) and thereby able to resist the forces of implantation. In addition, it is generally accepted that the majority of the growth factors most likely remain in the scaffold until the bleeding from the bone has ceased. Elution studies have shown a release profile of 50% release within 48 h, with growth factors detectable for several weeks thereafter. In contrast to the studies of fracture healing in a rat model (Schmidmaier et al. 2001b), there was no polymer left on our implants at the end of the observation period. The amount of growth factor used depends on the size of the implants, as the growth factors are administered in a specific w/w ratio to the poly(D,L-lactide) scaffold. The w/w ratio that we used was based on previous experience (Kandziora et al. 2003, Lamberg et al. 2006). It is possible that even better results would be obtained with different ratios, and this is the subject of further investigation.

### Interpretation

The mechanical fixation obtained with local release of TGF-β1 and IGF-1 is comparable to that of an HA coating. The mechanical fixation was achieved in different ways, as HA stimulated bone ongrowth while TGF -β1 and IGF-1 significantly increased gap healing. The results of this study suggest that controlled local growth factor release has the potential to be used in clinical situations in which a more general stimulation of bone is required, e.g. in revision arthroplasties or pseudoarthrosis. Given their different effects, a combination of the two coatings would be an interesting subject for further investigation. It is necessary to keep in mind that hydroxyapatite has had a long and successful history of clinical implementation, while growth factors may have unknown side effects when used clinically.

## References

[CIT0001] Capello WN, D'Antonio JA, Jaffe WL, Geesink RG, Manley MT, Feinberg JR. (2006). Hydroxyapatite-coated femoral components - 15-year minimum followup.. Clin Orthop.

[CIT0002] Dequeker J, Mohan S, Finkelman RD, Aerssens J, Baylink DJ. (1993). Generalized osteoarthritis associated with increased insulin-like growth factor types I and II and transforming growth factor beta in cortical bone from the iliac crest. Possible mechanism of increased bone density and protection against osteoporosis.. Arthritis Rheum.

[CIT0003] Eingartner C, Coerper S, Fritz J, Gaissmaier C, Koveker G, Weise K. (1999). Growth factors in distraction osteogenesis. Immuno-histological pattern of TGF-beta1 and IGF-I in human callus induced by distraction osteogenesis.. Int Orthop.

[CIT0004] Elmengaard B, Bechtold JE, Soballe K. (2005). In vivo effects of RGD-coated titanium implants inserted in two bone-gap models.. J Biomed Mater Res A.

[CIT0005] Gundersen HJ, Bendtsen TF, Korbo L, Marcussen N, Moller A, Nielsen K, Nyengaard JR, Pakkenberg B, Sorensen FB, Vesterby A. (1988). Some new, simple and efficient stereological methods and their use in pathological research and diagnosis.. APMIS.

[CIT0006] Hauptfleisch J, Glyn-Jones S, Beard DJ, Gill HS, Murray DW. (2006). The premature failure of the Charnley Elite-Plus stem: a confirmation of RSA predictions.. J Bone Joint Surg (Br).

[CIT0007] Kandziora F, Schmidmaier G, Schollmeier G, Bail H, Pflugmacher R, Gorke T, Wagner M, Raschke M, Mittlmeier T, Haas NP. (2002). IGF-I and TGF-beta1 application by a poly-(D,L-lactide)-coated cage promotes intervertebral bone matrix formation in the sheep cervical spine.. Spine.

[CIT0008] Kandziora F, Pflugmacher R, Scholz M, Schafer J, Schollmeier G, Schmidmaier G, Duda G, Raschke M, Haas NP. (2003). Dose-dependent effects of combined IGF-I and TGF-beta1 application in a sheep cervical spine fusion model.. Eur Spine J.

[CIT0009] Karrholm J, Borssen B, Lowenhielm G, Snorrason F. (1994). Does early micromotion of femoral stem prostheses matter? 4-7-year stereoradiographic follow-up of 84 cemented prostheses.. J Bone Joint Surg (Br).

[CIT0010] Kelly SJ, Incavo SJ, Beynnon B. (2006). The use of a hydroxyapatite-coated primary stem in revision total hip arthroplasty.. J Arthroplasty.

[CIT0011] Kveiborg M, Flyvbjerg A, Eriksen EF, Kassem M. (2001). Transforming growth factor-beta1 stimulates the production of insulin-like growth factor-I and insulin-like growth factor-binding protein-3 in human bone marrow stromal osteoblast progenitors.. J Endocrinol.

[CIT0012] Lamberg A, Schmidmaier G, Soballe K, Elmengaard B. (2006). Locally delivered TGF-beta 1 and IGF-1 enhance the fixation of titanium implants - A study in dogs.. Acta Orthopaedica.

[CIT0013] Lammens J, Liu Z, Aerssens J, Dequeker J, Fabry G. (1998). Distraction bone healing versus osteotomy healing: a comparative biochemical analysis.. J Bone Miner Res.

[CIT0014] Lind M, Overgaard S, Nguyen T, Ongpipattanakul B, Bunger C, Soballe K. (1996a). Transforming growth factor-beta stimulates bone ongrowth. Hydroxyapatite-coated implants studied in dogs.. Acta Orthop Scand.

[CIT0015] Lind M, Overgaard S, Ongpipattanakul B, Nguyen T, Bunger C, Soballe K. (1996b). Transforming growth factor-beta 1 stimulates bone ongrowth to weight-loaded tricalcium phosphate coated implants: an experimental study in dogs.. J Bone Joint Surg (Br).

[CIT0016] Nicolas V, Prewett A, Bettica P, Mohan S, Finkelman RD, Baylink DJ, Farley JR. (1994). Age-related decreases in insulin-like growth factor-I and transforming growth factor-beta in femoral cortical bone from both men and women: implications for bone loss with aging.. J Clin Endocrinol Metab.

[CIT0017] Overgaard S, Soballe K, Jorgen H, Gundersen G. (2000). Efficiency of systematic sampling in histomorphometric bone research illustrated by hydroxyapatite-coated implants: optimizing the stereological vertical-section design.. J Orthop Res.

[CIT0018] Pfeilschifter J, Erdmann J, Storch S, Ziegler R, Weinreb M. (1999). Changes in the concentration of insulin-like growth factor I and transforming growth factor beta1 in rat femoral bone during growth.. Calcif Tissue Int.

[CIT0019] Rose FR, Hou Q, Oreffo RO. (2004). Delivery systems for bone growth factors - the new players in skeletal regeneration.. J Pharm Pharmacol.

[CIT0020] Schmidmaier G, Wildemann B, Bail H, Lucke M, Fuchs T, Stemberger A, Flyvbjerg A, Haas NP, Raschke M. (2001a). Local application of growth factors (insulin-like growth factor-1 and transforming growth factor-beta1) from a biodegradable poly(D,L-lactide) coating of osteosynthetic implants accelerates fracture healing in rats.. Bone.

[CIT0021] Schmidmaier G, Wildemann B, Stemberger A, Haas NP, Raschke M. (2001b). Biodegradable poly(D,L-lactide) coating of implants for continuous release of growth factors.. J Biomed Mater Res.

[CIT0022] Schmidmaier G, Wildemann B, Gabelein T, Heeger J, Kandziora F, Haas NP, Raschke M. (2003). Synergistic effect of IGF-I and TGF-beta1 on fracture healing in rats: single versus combined application of IGF-I and TGF-beta1.. Acta Orthop Scand.

[CIT0023] Soballe K, Hansen ES, Brockstedt-Rasmussen H, Pedersen CM, Bunger C. (1990). Hydroxyapatite coating enhances fixation of porous coated implants. A comparison in dogs between press fit and noninterference fit.. Acta Orthop Scand.

[CIT0024] Soballe K, Hansen ES, Rasmussen H, Jorgensen PH, Bunger C. (1992). Tissue ingrowth into titanium and hydroxyapatite-coated implants during stable and unstable mechanical conditions.. J Orthop Res.

[CIT0025] Soballe K, Toksvig-Larsen S, Gelineck J, Fruensgaard S, Hansen ES, Ryd L, Lucht U, Bunger C. (1993). Migration of hydroxyapatite coated femoral prostheses. A Roentgen Stereophotogrammetric study.. J Bone Joint Surg (Br).

[CIT0026] Soballe K, Mouzin OR, Kidder LA, Overgaard S, Bechtold JE. (2003). The effects of hydroxyapatite coating and bone allograft on fixation of loaded experimental primary and revision implants.. Acta Orthop Scand.

[CIT0027] Soballe K, Jensen TB, Mouzin O, Kidder L, Bechtold JE. (2004). Differential effect of a bone morphogenetic protein-7 (OP-1) on primary and revision loaded, stable implants with allograft.. J Biomed Mater Res.

[CIT0028] Sumner DR, Turner TM, Urban RM, Virdi AS, Inoue N. (2006). Additive enhancement of implant fixation following combined treatment with rhTGF-beta2 and rhBMP-2 in a canine model.. J Bone Joint Surg (Am).

